# Glioma Cell Death Induced by Irradiation or Alkylating Agent Chemotherapy Is Independent of the Intrinsic Ceramide Pathway

**DOI:** 10.1371/journal.pone.0063527

**Published:** 2013-05-07

**Authors:** Dorothee Gramatzki, Caroline Herrmann, Caroline Happold, Katrin Anne Becker, Erich Gulbins, Michael Weller, Ghazaleh Tabatabai

**Affiliations:** 1 Laboratory of Molecular Neuro-Oncology, Department of Neurology, University Hospital Zurich, Zurich, Switzerland; 2 Department of Preclinical Imaging and Radiopharmacy, University Hospital Tuebingen, Tuebingen, Germany; 3 Department of Molecular Biology, University of Duisburg-Essen, Essen, Germany; 4 Neuroscience Center Zurich, University of Zurich and ETH Zurich, Zurich, Switzerland; Dresden University of Technology, Germany

## Abstract

**Background/Aims:**

Resistance to genotoxic therapy is a characteristic feature of glioma cells. Acid sphingomyelinase (ASM) hydrolyzes sphingomyelin to ceramide and glucosylceramide synthase (GCS) catalyzes ceramide metabolism. Increased ceramide levels have been suggested to enhance chemotherapy-induced death of cancer cells.

**Methods:**

Microarray and clinical data for ASM and GCS in astrocytomas WHO grade II–IV were acquired from the Rembrandt database. Moreover, the glioblastoma database of the Cancer Genome Atlas network (TCGA) was used for survival data of glioblastoma patients. For *in vitro* studies, increases in ceramide levels were achieved either by ASM overexpression or by the GCS inhibitor DL-threo-1-phenyl-2-palmitoylamino-3-morpholino-1-propanol (PPMP) in human glioma cell lines. Combinations of alkylating chemotherapy or irradiation and ASM overexpression, PPMP or exogenous ceramide were applied in parental cells. The anti-glioma effects were investigated by assessing proliferation, metabolic activity, viability and clonogenicity. Finally, viability and clonogenicity were assessed in temozolomide (TMZ)-resistant cells upon treatment with PPMP, exogenous ceramide, alkylating chemotherapy, irradiation or their combinations.

**Results:**

Interrogations from the Rembrandt and TCGA database showed a better survival of glioblastoma patients with low expression of ASM or GCS. ASM overexpression or PPMP treatment alone led to ceramide accumulation but did not enhance the anti-glioma activity of alkylating chemotherapy or irradiation. PPMP or exogenous ceramide induced acute cytotoxicity in glioblastoma cells. Combined treatments with chemotherapy or irradiation led to additive, but not synergistic effects. Finally, no synergy was found when TMZ-resistant cells were treated with exogenous ceramide or PPMP alone or in combination with TMZ or irradiation.

**Conclusion:**

Modulation of intrinsic glioma cell ceramide levels by ASM overexpression or GCS inhibition does not enhance the anti-glioma activity of alkylating chemotherapy or irradiation.

## Introduction

Glioblastoma is the most common primary malignant brain tumor [1]. Despite multimodal therapy the median overall survival does not exceed 11 months in population-based studies [2] or 15 months in selected clinical trial populations [3], [4]. The current standard of care for newly diagnosed glioblastoma includes radiotherapy (RT) with concomitant and maintenance temozolomide (TMZ) chemotherapy [5]. The nitrosoureas *N*-(2-chloroethyl)-*N'*-cyclohexyl-*N*-nitrosourea (CCNU) or *N,N'*-bis(2-chloroethyl)-*N*-nitrosourea (BCNU) are now commonly applied in recurrent glioblastoma [6], [7], [8]. A major therapeutic challenge remains the primary or acquired resistance of glioma cells to RT and/or alkylating agents, e.g. TMZ or CCNU. Therefore, new strategies to overcome the resistance to these therapeutic strategies are urgently needed.

Modulation of the sphingolipid signaling cascade had been suggested as a promising strategy to overcome resistance to anti-cancer therapies [9], [10]. Acid sphingomyelinase (ASM) is a glycoprotein localized in classical or secretory lysosomes that catalyses the hydrolysis of sphingomyelin to ceramide [11]. This results in the formation of ceramide-enriched membrane platforms and clustering of death receptors leading to apoptosis [11], [12]. ASM-induced ceramide accumulation induced apoptosis in response to the activation of pro-apoptotic receptor proteins, e.g. cluster of differentiation 95 (CD95) [13], [14], [15] or tumor necrosis factor receptor [16]. Several stress stimuli can also activate ASM signaling, e.g. irradiation [17], [18], ultraviolet light [19] and chemotherapeutic drugs. Smith and Schuchman analyzed the microarray database Oncomine (www.oncomine.org) in 2008 and concluded that in some cancers ASM may be down-regulated, causing reduced ceramide levels [9]. Chemotherapy-induced ASM activation and apoptosis were demonstrated in human colon cancer cell lines after cisplatin therapy [20], in human ovarian cancer cells upon paclitaxel therapy [21] and in neuroblastoma cells upon fenretinide treatment [22]. ASM overexpression sensitized the murine glioma cell line GL-261 and the human glioma cell line U373MG to gemcitabine or doxorubicin [23]. ASM expression in U87MG glioma cells was found to be p53-independent and sensitized these cells to irradiation [24].

Ceramide levels are low in several tumor types compared to non-maligant tissue, including colon cancer [25] and ovarian cancer [26]. Riboni et al. suggested that ceramide levels decrease during malignant progression of human gliomas [27]. Recently the same group proposed that TMZ increases endogenous ceramide in human glioblastoma cell lines *in vitro* but not in TMZ-resistant cells [28]. We previously demonstrated that exogenous C2-ceramide induced apoptosis in human glioma cell lines and that the combination of C2-ceramide and CD95L induced cell death synergistically in T98G and LNT-229 glioma cells [29].

Overexpression of glucosylceramide synthase (GCS), an enzyme leading to ceramide degradation, enhanced resistance to doxorubicin in breast cancer cell lines. Inhibitors of GCS restored sensitivity of these cells to chemotherapy [30], [31]. The inhibition of GCS also sensitized mouse glioma cells to gemcitabine [32]. Similar results were published for TMZ-resistant human glioblastoma cells [28]. Synergistic effects of GCS inhibition and chemotherapeutic drugs were also demonstrated for neuroblastoma, melanoma, prostate, lung, colon and pancreatic cancer [33], [34]. Moreover, overexpression of GCS was found in chemoresistant leukemia cells [35]. On the other hand, several groups defined limitations of the role of GCS for resistance to cancer chemotherapy [36], [37], [38].

Based on these data, we investigated the impact of modulating endogenous ceramide levels on the resistance to clinically relevant therapies at clinically relevant concentrations respectively doses in LNT-229 and T98G human glioma cells lines *in vitro*. Modulations of the endogenous ceramide pathway were achieved either by stable ASM overexpression or by inhibition of GCS using the specific inhibitor DL-threo-1-phenyl-2-palmitoylamino-3-consumption (PPMP) [39]. Finally exogenous ceramide analogs were used, too. As treatment modalities, we used clinically relevant concentrations of alkylating agents, i.e. TMZ and CCNU, as well as clinically relevant doses of irradiation.

## Materials and Methods

### Cells and reagents

The human malignant glioma cell line T98G was obtained from the American Type Culture Collection (Rockville, MD). LNT-229 cells and LN-18 cells were kindly provided by N. de Tribolet (Lausanne, Switzerland) and have been used in previous studies of our laboratory [40]. TMZ-resistant cells were generated by repetitive exposure of LNT-229 and LN-18 glioma cells to TMZ. The TMZ-resistant cells are referred to as LN-18_R and LNT-229_R and have been characterized in our laboratory [41]. The cells were maintained in Dulbecco's modified eagle medium (DMEM) containing 10% fetal calf serum (FCS) (Biochrom KG, Berlin, Germany) and 2 mM glutamine and penicillin/streptomycin. A monoclonal antibody to human ASM suitable for immunoblotting was purchased from Cell Signaling Technology (Boston, MA, catalogue number 3687, source rabbit). The antibody to β-actin was obtained from Santa Cruz Biotechnology (Santa Cruz, CA, catalogue number sc-1616-R, source rabbit) and the antibody to glyceraldehyde 3-phosphate dehydrogenase (GAPDH) was obtained from Everest Biotech (Ramona, CA, USA, catalogue number EB06377). The GCS antibody was purchased from Abcam (Cambridge, United Kingdom, catalogue number 3687, ab98030, source rabbit). TMZ was obtained from Schering-Plough (Kenilworth, NJ, USA) and stock solutions were prepared in dimethylsulfoxide. C2-ceramide (N-acetyl-D-sphingosine), C6-ceramide (N-hexanoyl-D-sphingosine), PPMP (DL-threo-1-Phenyl-2-palmitoylamino-3-morpholino-1-propanol) and ami-triptyline were purchased from Sigma-Aldrich (St. Louis, MO, USA).

### Lentiviral production, titration and transduction

A lentiviral backbone containing an enhancing green fluorescent protein (eGFP) cassette driven by an internal spleen focus forming virus (SFFV) promoter and containing an additional pIRES sequence, lenti-SIEW (spleen focus forming virus promoter, pIRES, eGFP, WPRE), was kindly provided by Manuel Grez [42]. The ASM sequence was cloned into lenti-SIEW. Both strands of the final pSIEW-ASM were analyzed to verify the correct DNA sequence. Lentivirus was produced as previously described [43]. Briefly, the lentivirus was generated by co-transfecting 293T cells with the vector and the packaging constructs. Supernatants of these cells, containing the lentivirus, were collected 48 and 72 hours (h) after co-transfection. Supernatants were concentrated by ultracentrifugation and used directly to transduce target cells with 100 transducing units per ml in 24-well plates.

### Real-time polymerase chain reaction (RT-PCR)

Total RNA was prepared using the NucleoSpin System (Macherey-Nagel AG, Oensingen, Switzerland) and transcribed according to standard protocols. cDNA was prepared using Superscript RNase H reverse transcriptase (Invitrogen, Paisley, UK) and random hexamers (Sigma-Aldrich). For RT-PCR, cDNA amplification was measured using the 7300 Real-time PCR System (Applied Biosystems, Zug, Switzerland) with SYBR Green Master Mix (Eurogentec, Cologne, Germany) and primers (Metabion, Martinsried, Germany) at optimized concentrations. ADP-ribosylation factor 1 (ARF1) or GAPDH were used as housekeeping genes [44]. The following primers were used: ARF1, forward 5′-GAC CAC GAT CCT CTA CAA GC-3′, reverse 5′-TCC CAC ACA GTG AAG CTG ATG-3′; GAPDH, forward 5′-CTC TCT GCT CCT CCT GTT CGA C-3′, reverse 5′-TGA GCG ATG TGG CTC GGC T-3′; ASM, forward 5′-TAC ATC GCA TAG TGC CCC GGC T-3′, reverse 5′-CCC ACG CGA GCC ACA TTG GGT-3′; GCS, forward 5′-ATG ACA GAA AAA GTA GGC TTG G-3′, reverse 5′-GGA CAC CCC TGA GTT GAA-3′. Relative quantification of gene expression was determined by comparison of threshold values. All results were normalized to ARF1 and calculated with the ΔC_TT_ method for relative quantification [45].

### Immunoblot analysis

For the detection of proteins from cell lysates, cells were lysed in radioimmunoprecipitation assay buffer (10 mM Tris pH 8.0, 150 mM NaCl, 1% NP-40, 0.5% deoxycholate, 0.1% sodium dodecyl sulphate) supplemented with 1×complete inhibitor mix (Roche Diagnostics, Grenzach-Wyhlen, Germany), and phosphatase inhibitor cocktails 1 and 2 (Sigma-Aldrich). Protein concentrations were measured with the Bradford protein assay reagent (Bio-Rad Laboratories, Hercules, California, USA) with bovine serum albumin as a standard. Protein levels were analyzed by immunoblot using 20 µg of protein per lane mixed with Laemmli buffer containing β-mercaptoethanol unless otherwise indicated with the respective antibodies in the concentrations recommended by the manufacturer. Equal protein loading was ascertained by Ponceau S (Sigma Aldrich) staining. Visualization of protein bands was accomplished using horseradish peroxidase-coupled secondary antibodies (Santa Cruz) and enhanced chemiluminescence (Perbio, Bonn, Germany).

### Acute cytotoxicity assay

Five thousand cells were seeded per well in 96-well plates and allowed to attach for 24 h. The cells were treated with the respective agent for 24 h under serum-free conditions. 72 h later the cell culture medium was removed and surviving cells were stained with crystal violet and optical density values were read in an ELISA reader at 560 nm wavelength.

### Clonogenic survival assay

Five hundred cells were seeded per well in 6-well plates in DMEM/10% FCS. Medium was removed after 24 h and cells were treated in serum-free medium with the respective reagents for 24 h and then kept in medium supplemented with 10% FCS. When clones were detected by microscopy, the medium was removed, and the number of colonies was quantified using crystal violet staining.

### Cell proliferation assay

Proliferation assays were performed as previously described [46]. In brief, 1.000 cells were seeded per well (24-well pate) in DMEM/10% FCS. Cells were counted every 24 h up to 7 days using trypan blue staining.

### Alamar Blue assay

Metabolic activity was assessed using the Alamar Blue Assay (Invitrogen). 10.000 cells were seeded per well (96-well plate) in DMEM/10% FCS. 24 h later the mix-and-ready Alamar Blue Solution was added in each well for 4 h. Absorbance was measured at 570 nm. The amount of absorbance is proportional to the number of living cells and corresponds to the cellular metabolic activity [47].

### Gene expression analysis and Kaplan Meier analysis of survival probability using Rembrandt database and the Cancer Genome Atlas network

Microarray and clinical data were obtained from the Repository for Molecular BRAinNeoplasiaDaTa (Rembrandt) using data available on August 04, 2011 (https://caintegrator.nci.nih.gov/rembrandt/) [48] and from the glioblastoma data set of the Cancer Genome Atlas network available on December 01, 2012 (http://cancergenome.nih.gov/) [49]. Gene expression data in both glioma databases were collected using Affymetrix® gene chips. The query in both databases was based on the reporter with the highest mean geometric intensity for the target gene (ASM: 209420_s_at; GCS: 204881_s_at). The Rembrandt database provides data of patients diagnosed for astrocytoma WHO grade II–IV. The group of glioblastoma patients in this database includes 228 cases. In addition, we also used the TCGA database and analyzed data from 504 glioblastoma patients.

Gene expression and Kaplan-Meier survival data for ASM or GCS were queried following the Rembrandt site's instructions for “advanced search” and via the caINTEGRATOR homepage (https://caintegrator2.nci.nih.gov) following the site's instructions. The sample group for gene expression was restricted to astrocytomas WHO grade II/III (n = 148) and glioblastomas (n = 228) and compared to normal brain tissues (n = 28). Survival data for the Kaplan-Meier analysis using the Rembrandt database were available from patients diagnosed with astrocytoma WHO grade II/III (n = 162) and glioblastoma (n = 181). Samples with a 2-fold up-regulation or a 2-fold down-regulation of the target gene compared to normal brain tumor tissue were defined as up- or down-regulated, the other samples were defined as intermediate.

Survival analysis within the glioblastoma data set of the TCGA database (n = 504) was performed using the Kaplan-Meier analysis module of the R2 microarray analysis and visualization platform (http://r2.amc.nl). The averaged mRNA expression staining for ASM was scaled to 65 for ASM and 299 for GCS. The cut-off for the highest impact on survival was 39.3 for ASM and 216 for GCS. This cut-off divides the glioblastoma patients in two groups with high or low expression of the target gene.

### ASM activity

ASM activity was determined as described [23]. Briefly, 200,000 cells were seeded per well (6-well plate) and allowed to attach for 24 h. Cells were lysed in a buffer consisting of 250 mM sodium acetate (pH 5.0), 1% NP-40, 1.3 mM ethylenediaminetetraacetic acid (EDTA), diluted to 0.1% NP40, and incubated with 50 nCi [^14^C]-sphingomyelin per sample (Perkin Elmer, Waltham, MA, USA; 52 mCi/mmol) for 30 min at 37°C. The substrate was dried prior to the assay, resuspended in 250 mM sodium acetate (pH 5.0), 0.1% NP-40, 1.3 mM EDTA and sonicated for 10 min in a bath sonicator to obtain micelles. The reaction was stopped by the addition of 800 µL chloroform/methanol (2∶1, v/v), and phases were separated by centrifugation. Radioactivity of the aqueous phase was measured by using liquid scintillation counting to determine the release of [^14^C]phosphorylcholine from [^14^C]sphingomyelin as a measure of ASM activity.

### Ceramide measurements

Ceramide concentrations were determined by DAG kinase assays as described [50]. Glioma cells were treated with PPMP, irradiation or TMZ. Medium was removed 12 h later and the cells were lysed in 300 microliter methanol, homogenized by tip sonication, brought to CHCl_3_:CH_3_OH:1N HCl (100∶100∶1, v/v/v) and 200 µL H_2_O were added. The samples were vortexed, the phases were separated, the lower phase was collected, dried, resuspended in 20 µL of a detergent solution (7.5% [w/v] n-octyl glucopyranoside, 5 mM cardiolipin in 1 mM diethylenetriaminepentaacetic acid [DTPA]), and sonicated for 10 min. The kinase reaction was started by the addition of 70 µL of a reaction mixture containing 10 µL diacylglycerol (DAG) kinase (GE Healthcare Europe, Munich, Germany), 0.1 M imidazole/HCl (pH 6.6), 0.2 mM DTPA (pH 6.6), 70 mM NaCl, 17 mM MgCl_2_, 1.4 mM ethylene glycol tetraacetic acid, 2 mM dithiothreitol, 1 µM adenosine triphosphate (ATP), and 5 µCi [^32^P]γATP. The kinase reaction was performed for 30 min at room temperature and terminated by the addition of 1 mL CHCl_3_:CH_3_OH:1N HCl (100∶100∶1, v/v/v), 170 µL buffered saline solution (135 mM NaCl, 1.5 mM CaCl_2_, 0.5 mM MgCl_2_, 5.6 mM glucose, 10 mM HEPES [pH 7.2]), and 30 µL of a 100 mM EDTA solution. The lower phase was collected, dried, and separated on Silica G60 thin-layer chromatography (TLC) plates with chloroform/acetone/methanol/acetic acid/H_2_O (50∶20:15:10:5, v/v/v/v/v). The TLC plates were exposed to radiography films, the spots were removed from the plates, and the incorporation of [^32^P] into ceramide was measured by liquid scintillation counting. Ceramide amounts were determined by comparison with a standard curve using C16 to C24 ceramides as substrates. Ceramide amounts were normalized to protein concentrations.

### Statistics

Analysis of significance was performed using the two-tailed Student's *t*-test (*t*-test) (Excel, Microsoft, Redmond, Washington, USA). For Kaplan-Meier survival analysis the log-rank test was used. All experiments were done at least three times and at least one representative experiment is shown. To study if co-treatments show additive or synergistic results, the fractional product method was used. The effect of two independently acting agents is defined as the product of the unaffected fractions after treatment with either agent alone, named the predicted value. If the observed value of the co-treatment exceeds that of the calculated product, the two agents show synergy [51], [52].

## Results

### ASM and GCS expression and clinical outcome in glioma patients

In this study we modified the activity of two proteins, ASM and GCS, with the aim to increase endogenous ceramide levels in human glioma cells *in vitro* and to investigate the impact of intrinsic ceramide levels on resistance to TMZ, CCNU or irradiation. First, we explored the potential role of these two genes in glioma patients using the Rembrandt and TCGA databases.

**Figure 1:**
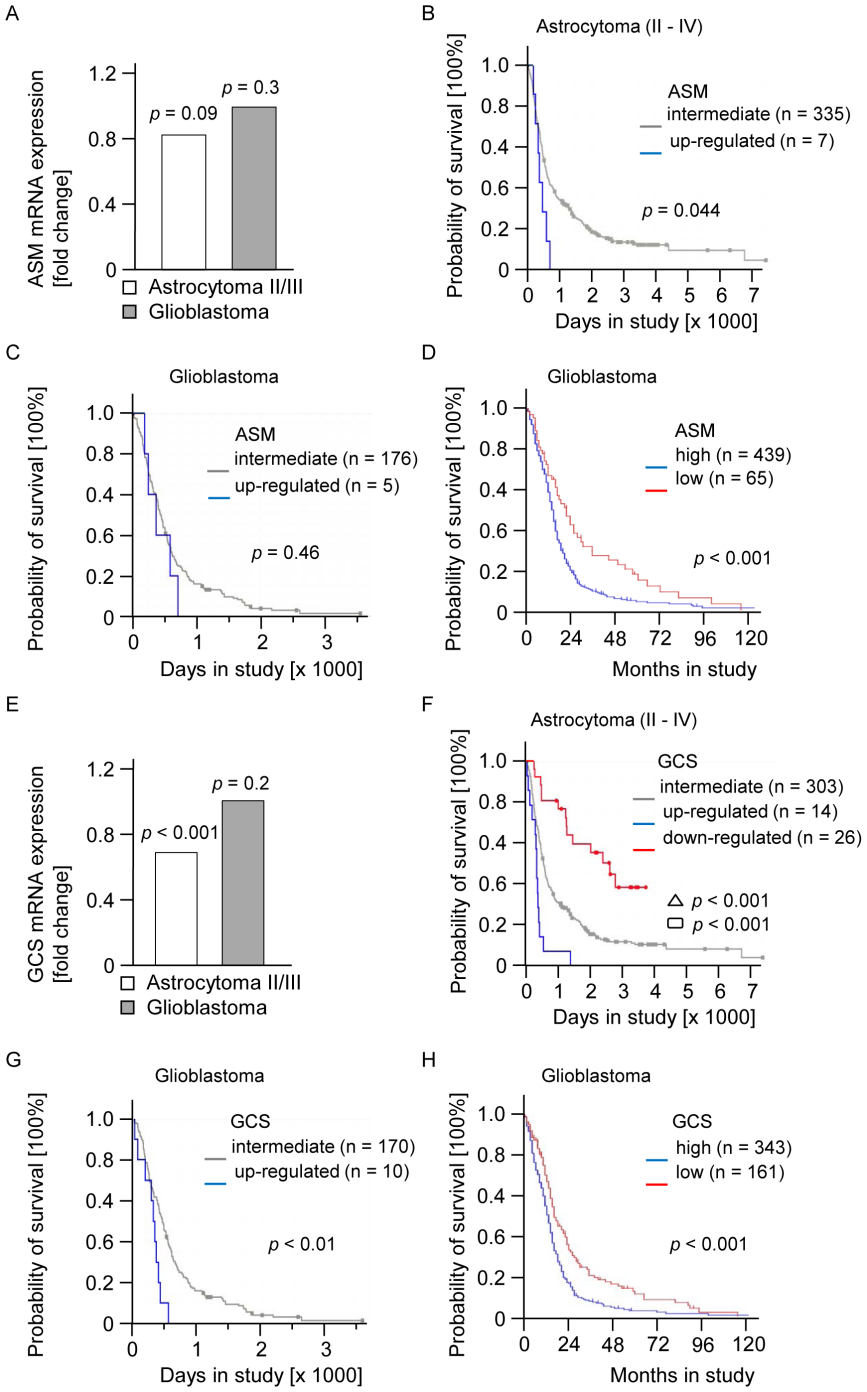
ASM and GCS expression and clinical outcome in glioma patients. (A) ASM mRNA expression was assessed in astrocytomas (WHO grades II/III) and glioblastomas, normalized to normal brain (Rembrandt database). Survival probabilities of patients with (B) astrocytoma (WHO grades II–IV) and (C) glioblastoma with at least 2-fold up-regulation of ASM (blue) compared with those of patients with intermediate expression of ASM (grey) (Rembrandt database). (D) Survival probabilities of patients with glioblastoma with high expression of ASM (blue) compared with those of patients with low expression of ASM (red) (TCGA database). (E) GCS mRNA expression of astrocytomas (WHO grades II/III) and glioblastomas, normalized to normal brain (Rembrandt database). Survival probabilities of patients with (F) astrocytoma (WHO grades II–IV) and (G) glioblastoma with at least 2-fold up-regulation of GCS (blue) or at least 2-fold down-regulation of GCS (red) (only in F) compared to those of patients with intermediate expression of GCS (grey) (Rembrandt database). (H) Survival probabilities of patients with glioblastoma with high expression of GCS (blue) compared with those of patients with low expression of GCS (red) (TCGA database). *p*-values were assessed using the log-rank test (*p*<0.05 was considered significant).

First we analyzed the mRNA expression of ASM in glioma patients in the Rembrandt database, showing that ASM mRNA levels did not differ in human glioblastomas or astrocytomas WHO grade II/III compared to normal brain (Fig. 1A). Interestingly, the survival analysis revealed that the overall survival of patients with glioma (WHO grades II–IV) with a more than 2-fold increase of ASM was reduced in comparison with patients with intermediate expression, but this analysis is limited by the fact that only 7 patients showed increased levels of ASM mRNA (Fig. 1B). A downregulation of ASM mRNA more than 2-fold, on the other hand, was not detected in the Rembrandt database. Next, we analyzed the clinical outcome data in glioblastoma patients in the Rembrandt database. Five patients showed a more than 2-fold ASM increase compared to normal brain tissue without any correlation to the probability of survival (Fig. 1C). Therefore, we investigated a larger group of glioblastoma patients and analyzed the TCGA database for a statistically ideal cut-off, dividing the group of glioblastoma patients in patients with a high and patients with a low expression of ASM. Kaplan-Meier curves derived from the TCGA database demonstrated longer survival of glioblastoma patients with low levels of ASM (Fig. 1D). Taken together, interrogations of the Rembrandt and TCGA database did not establish a strong correlation between loss of ASM expression and poor outcome.

Next, we assessed the gene expression of GCS in the same samples of glioma patients in the Rembrandt database, showing that GCS mRNA expression was not differentially regulated in human glioblastoma tissues compared to normal brain (Fig. 1E). In the sample group of astrocytomas grade II/III GCS mRNA levels were significantly lower than in glioblastoma tissue but also compared to normal brain tissue. Analysis of the Rembrandt database revealed that the overall survival of patients with glioma (WHO grades II–IV) with a more than 2-fold increase of GCS was significantly reduced and with a more than 2-fold decrease of GCS significantly prolonged in comparison with patients with intermediate expression of this gene (Fig. 1F). Regarding GCS expression in the group of glioblastoma patients, only a small group of 10 patients showed a more than 2-fold GCS upregulation, associated with a significant decrease in probability of survival, whereas only one patient was found in the Rembrandt database showing a more than 2-fold reduced GCS mRNA expression. In line with these data, analysis of the TCGA database revealed that in patients with glioblastoma, high expression of the GCS gene was associated with decreased overall survival (Fig. 1H). Taken together, these data suggest a correlation between high GCS expression and early death.

### Stable overexpression of ASM increases ceramide levels in human glioma cell lines, but does not alter proliferation, metabolic activity or clonogenicity

**Figure 2:**
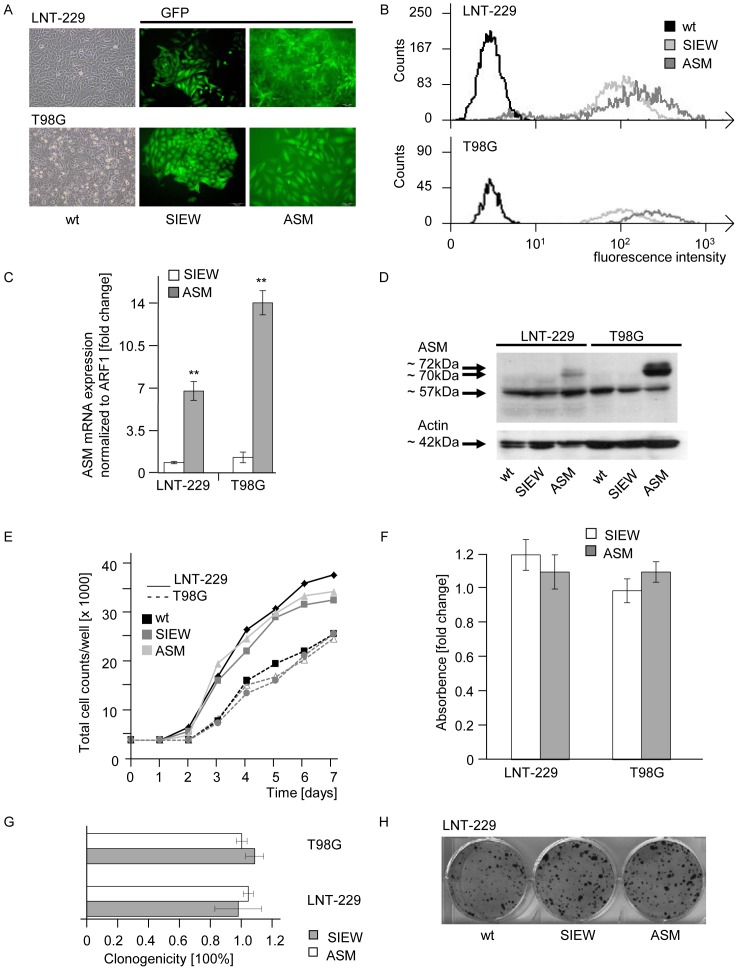
Stable overexpression of ASM increases ceramide levels in human glioma cell lines, but does not alter proliferation, metabolic activity or clonogenicity. Assessment of lentiviral transduction efficacy by (A) fluorescence microscopy and (B) flow cytometry; *wt*, wild-type cells; *SIEW*, control-transduced cells; *ASM*, ASM-overexpressing cells. (C) ASM mRNA expression levels (statistically significant changes are marked with asterisks; **, *p*<0.01; *t*-test) and (D) protein levels were assessed in wt, SIEW or ASM glioma cells. (E) Proliferation of LNT-229 or T98G wt, SIEW and ASM cells was measured up to 7 days by counting viable cells with trypan blue staining. One experiment out of three independent experiments is shown, all giving similar results. (F) Metabolic activity in these cell lines was assessed by Alamar Blue staining. (G) Clonogenicity is presented for LNT-229 and T98G SIEW and ASM cells, normalized to wt cells. (H) One representative sample from (G) is shown for LNT-229 wt, SIEW control transduced and ASM overexpressing cells.

The two human glioma cell lines LNT-229 and T98G were stably lentivirally transduced with SIEW-ASM or control virus. The lentiviral transduction efficacy with the GFP-positive SIEW construct was confirmed by fluorescence microscopy (Fig. 2A) and flow cytometry (Fig. 2B). Real-time PCR showed a strong increase of ASM mRNA levels in both ASM-overexpressing cell lines, LNT-229-SIEW-ASM and T98G-SIEW-ASM. The increase of ASM mRNA was up to 7-fold in LNT-229 and 14-fold in T98G (Fig. 2C). ASM protein levels were determined by immunoblot (Fig. 2D). Three forms of ASM are detectable, at 72 kDa, 70 kDa and 57 kDa. The 57 kDa form is probably generated by proteolytic cleavage inside the endoplasmatic reticulum/Golgi complex whereas the lysosomal predominant mature 70 kDa form, processed from a 72 kDa precursor form, is exclusively processed inside acidic organelles [53]. The 72 and 70 kDa forms were dominantly detected in ASM-overexpressing cells, whereas the 57 kDa form was seen in all transduced and non-transduced cell lines and was even decreased in T98G-SIEW-ASM (Fig. 2D). ASM protein levels for the 70 kDa form did not change in LNT-229-SIEW-ASM. ASM overexpression resulted in increased ASM activity, an induction of 1.9-fold in LNT-229 and 5.4-fold in T98G, measured as release of phosphorylcholine from sphingomyelin, was observed. ASM overexpression led to an increase of intracellular endogenous ceramide levels, up to 3-fold in LNT-229 and 2.3-fold in T98G. ASM protein overexpression alone had no effect on proliferation (Fig. 2E), metabolic activity (Fig. 2F) or clonogenicity (Fig. 2G, H) in either cell line.

### ASM overexpression does not sensitize glioma cells to alkylating agents or irradiation in vitro

**Figure 3:**
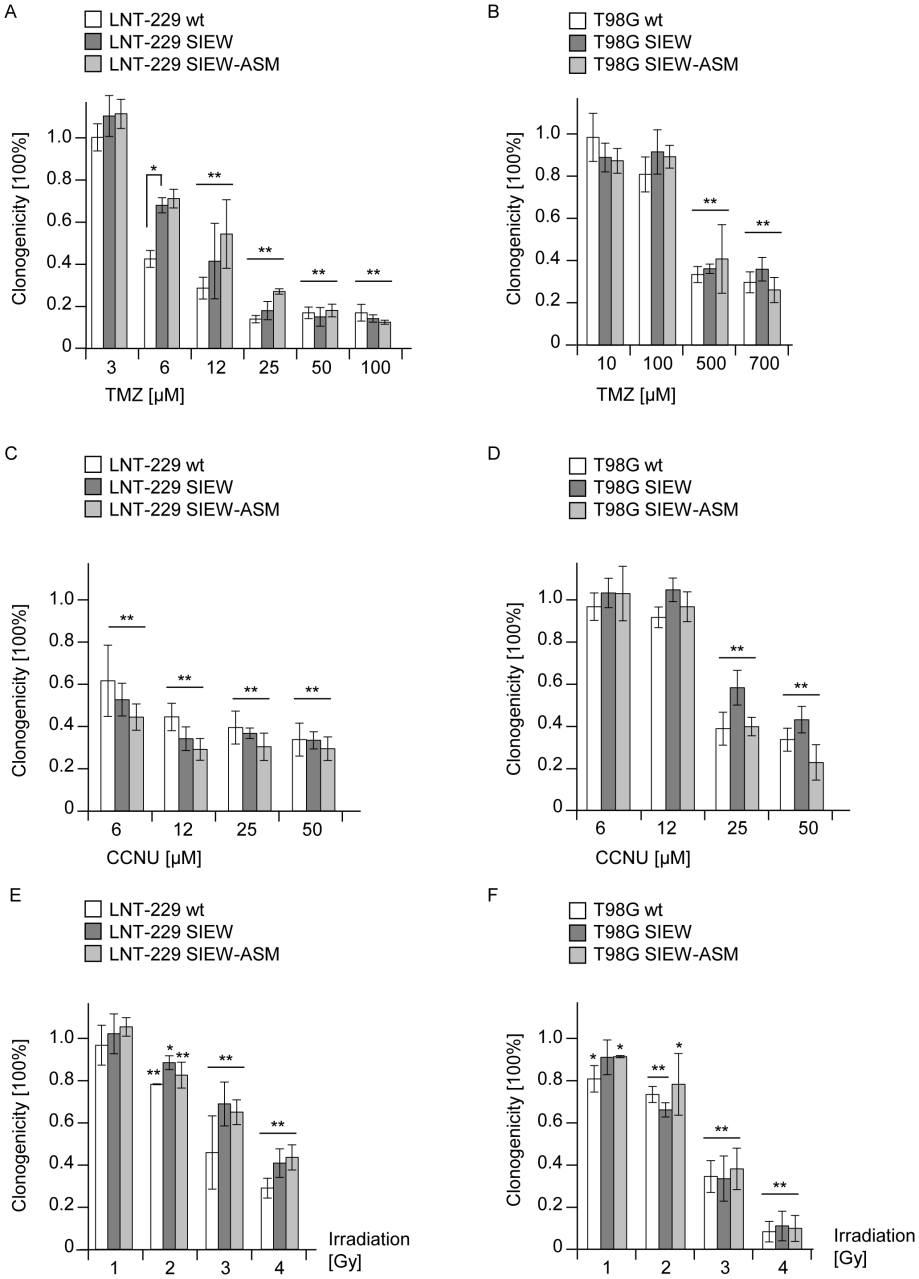
ASM overexpression does not sensitize glioma cells to alkylating agents or irradiation in vitro. Human glioma wt (open bars), SIEW (dark grey bars) or ASM (light grey bars) cells of (A, C, E) LNT-229 or (B, D, F) T98G were treated with increasing concentrations of (A, B) TMZ or (C, D) CCNU for 24 h or increasing doses of (E, F) irradiation. Statistically significant changes compared to untreated cells are marked with asterisks (*, *p*<0.05; **, *p*<0.01; *t*-test).

We treated LNT-229 (Fig. 3A, C) or T98G (Fig. 3B, D) cells for 24 h with increasing concentrations of TMZ (Fig. 3A, B) or CCNU (Fig. 3C, D). The medium was removed and cells cultured with new medium for up to 2 weeks. As expected [54], [55], both drugs, TMZ and CCNU, reduced the clonogenic survival in a concentration-dependent manner (Fig. 3A–D). ASM overexpression did not affect the clonogenic survival of glioma cells co-exposed to TMZ or CCNU. Moreover ASM-overexpressing LNT-229 and T98G showed unaltered sensitivity to irradiation up to 4 Gy (Gray) (Fig. 3E, F). Assuming that a sensitizing effect of ASM overexpression correlates to increased ceramide levels in response to chemo- or radiotherapy, ceramide levels after treatment with TMZ or irradiation were assessed. Irradiation increased ceramide levels up to 1.7-fold in LNT-229 and up to 1.5-fold in T98G. TMZ treatment induced ceramide accumulation in both cell lines, LNT-229 (2.1-fold) and T98-G (2.6-fold). To exclude a very early selection step after ASM overexpression promoting the survival of cells resistant to ASM activity, ASM was overexpressed in LNT-229 in the presence or absence of amitriptyline (final concentration 2.5 µM), an ASM inhibitor. Amitriptyline treatment was stopped 24 h before the onset of experiments. Yet, ASM overexpression did not affect proliferation or clonogenicity under these conditions either (data not shown).

### PPMP induces acute cytotoxicity and reduces clonogenic survival in glioma cell lines

**Figure 4:**
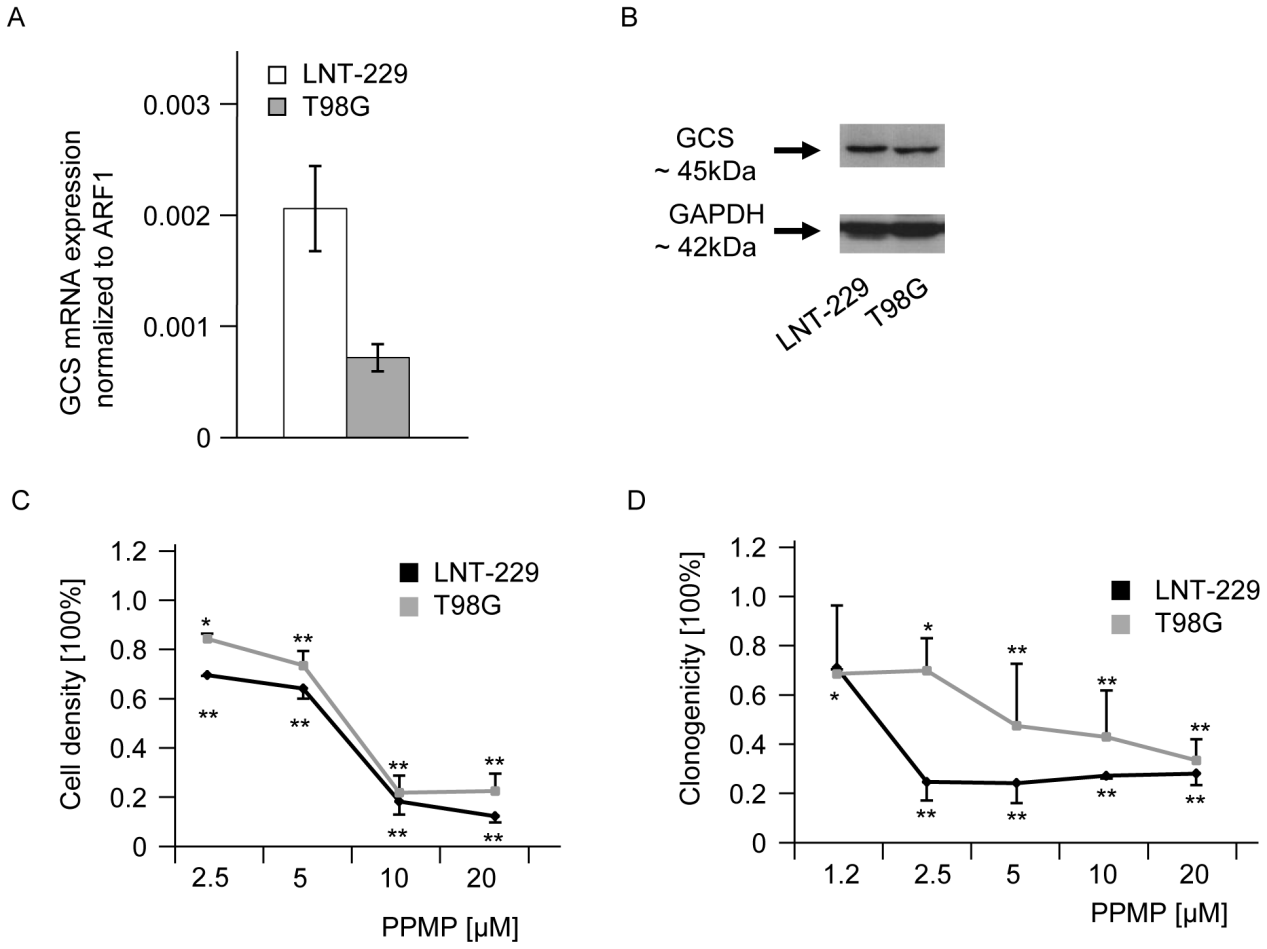
PPMP reduces acute cytotoxicity and clonogenic survival in human glioma cell lines. (A) GCS mRNA and (B) protein expression were assessed in LNT-229 and T98G human glioma cells. ARF1 was used as housekeeping gene for mRNA measurements and GAPDH for immunoblot analyses. (C) Acute cytotoxicity was assessed 72 h after treatment with increasing concentrations of PPMP in these glioma cells. (D) For clonogenic survival data LNT-229 or T98G cells were treated with increasing concentrations of PPMP for 24 h, the medium was changed and cells were cultured for additional 2 weeks until colonies were detectable. Statistically significant changes compared to untreated cells are marked with asterisks (*, *p*<0.05; **, *p*<0.01; *t*-test).

A second strategy to increase endogenous ceramide levels was the application of PPMP, an inhibitor of GCS, described to sensitize different tumor types to chemotherapy or irradiation. First we confirmed that the two glioblastoma cell lines LNT-229 and T98G express the enzyme GCS as assessed on mRNA (Fig. 4A) and protein level (Fig. 4B). To confirm the biological activity of PPMP, endogenous ceramide levels were measured 12 h after treatment with 1 or 10 µM PPMP. In both glioma cell lines ceramide levels increased in a concentration-dependent manner up to 2.5–2.8 fold (data not shown). The effect of PPMP on glioma cell viability was determined in acute cytotoxicity and clonogenicity assays. PPMP induced acute cytotoxicity in a concentration-dependent manner and the half-maximal inhibitory concentration (IC50) values for PPMP were between 5 and 10 µM (72 h treatment) (Fig. 4C). When glioblastoma cells were treated with PPMP in a clonogenic survival assay for up to 3 weeks, concentrations between 1 and 2 µM in LNT-229 and between 5 and 10 µM in T98G reduced clonogenic survival up to 50% (Fig. 4D).

### Co-treatment with PPMP and TMZ or irradiation shows additive but not synergistic effects in vitro

**Figure 5:**
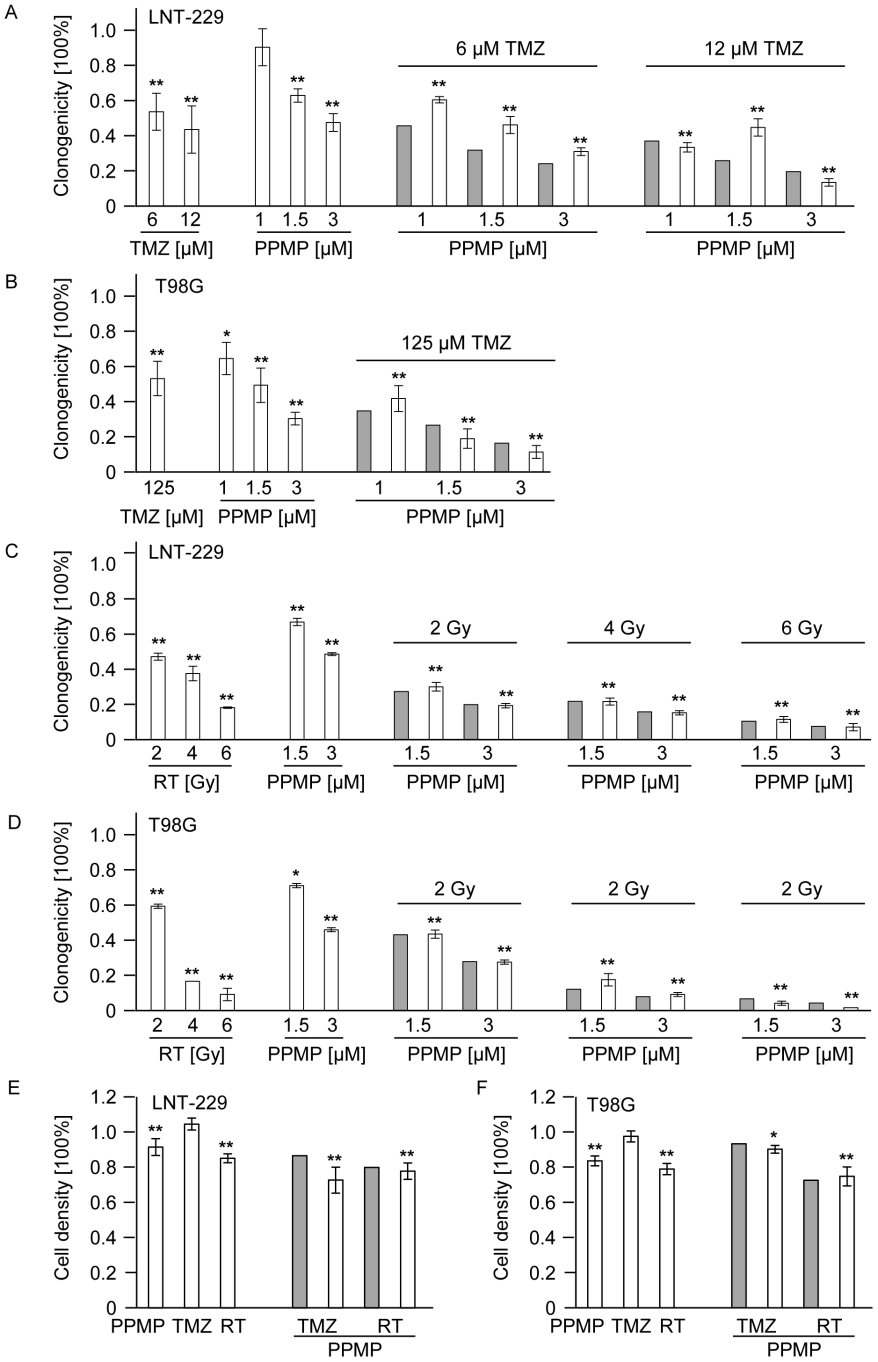
Co-treatment with PPMP and TMZ or irradiation shows additive but not synergistic effects in vitro. (A) LNT-229 or (B) T98G wt cells were treated with increasing concentrations of TMZ or PPMP or co-treated with both agents for 24 h and clonogenicity assessed 2–3 weeks later. (C) LNT-229 or (D) T98G wild-tye cells were treated with increasing concentrations of PPMP for 24 h and increasing doses of RT or co-treated with PPMP and irradiation and clonogenicity data obtained 2–3 weeks later. (E) LNT-229 or (F) T98G wt cells were treated with a single dose of PPMP (5 µM) or TMZ (LNT-229: 12 µM; T98G: 125 µM) for 72 h or RT (8 Gy) or co-treated with PPMP and TMZ or RT and cell density measured with crystal violet 72 h later. Predicted values, assessed using the fractional product method, are displayed in grey bars, observed values are highlighted by blank bars. Statistically significant changes compared to untreated cells are marked with asterisks (*, *p*<0.05; **, *p*<0.01; *t*-test).

Clonogenic survival data for LNT-229 (Fig. 5A, C) and T98G (Fig. 5B, D) were obtained for treatments with TMZ, PPMP or irradiation alone as well as for co-treatments with TMZ and PPMP (Fig. 5A, B) or irradiation and PPMP (Fig. 5C, D). The fractional product method was used to define predicted values in the case of independent actions of PPMP and TMZ or irradiation, respectively. These predicted values were compared with the actual clonogenic survival. Treatments of TMZ, PPMP or irradiation alone reduced clonogenic survival in a concentration-/dose-dependent manner. When co-treatments were used, however, the observed values exactly matched the predicted values for independent actions. Thus, the combination of PPMP with TMZ or irradiation led to additive rather than synergistic effects in both glioma cell lines in a clonogenic survival assay. To be sure that sensitizing effects of PPMP and irradiation or TMZ do not occur in the first days of treatment and may not be detectable in the clonogenic survival assays later, acute cytotoxicity assays were performed in addition. Again, no synergistic effects of PPMP in combination with TMZ or irradiation were observed in LNT-229 (Fig. 5E) and T98G (Fig. 5F) parental cells.

### Exogenous ceramide induces cytotoxicity and reduces clonogenicity in human glioma cell lines, but not synergistically in combination with irradiation or TMZ

**Figure 6:**
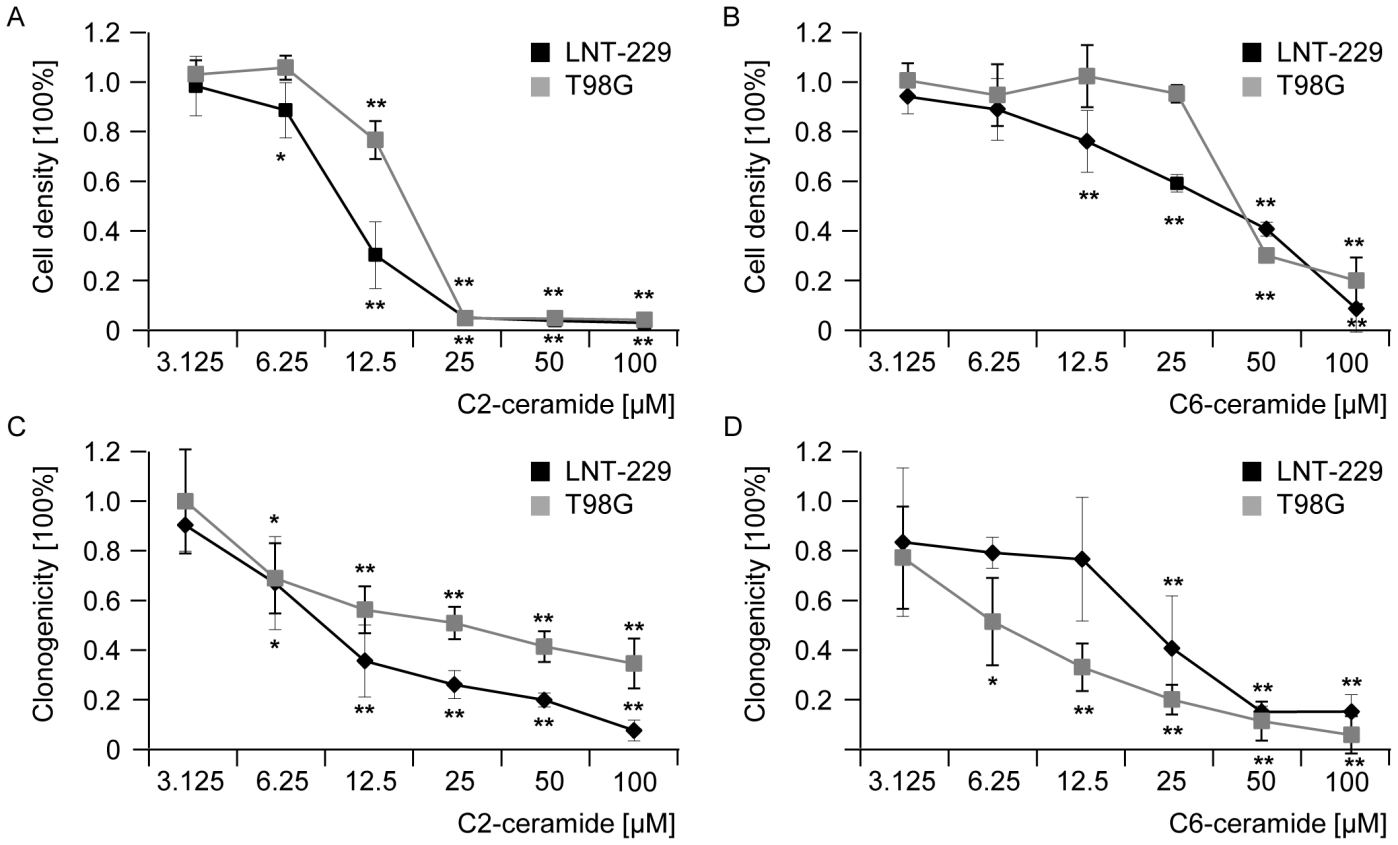
Exogenous ceramide is cytotoxic to human glioma cell lines and reduces clonogenic survival. Acute cytotoxicity was assessed 72 h after treatment with increasing concentrations of (A) C2-ceramide or (B) C6-ceramide in LNT-229 or T98G cells. For clonogenic survival data LNT-229 or T98G cells were treated with increasing concentrations of (C) C2-ceramide or (D) C6-ceramide for 24 h, then medium was changed and cells were cultured for additional 2 weeks until colonies were detectable. *, *p*<0.05; **, *p*<0.01; compared to untreated cells; *t*-test.

**Figure 7:**
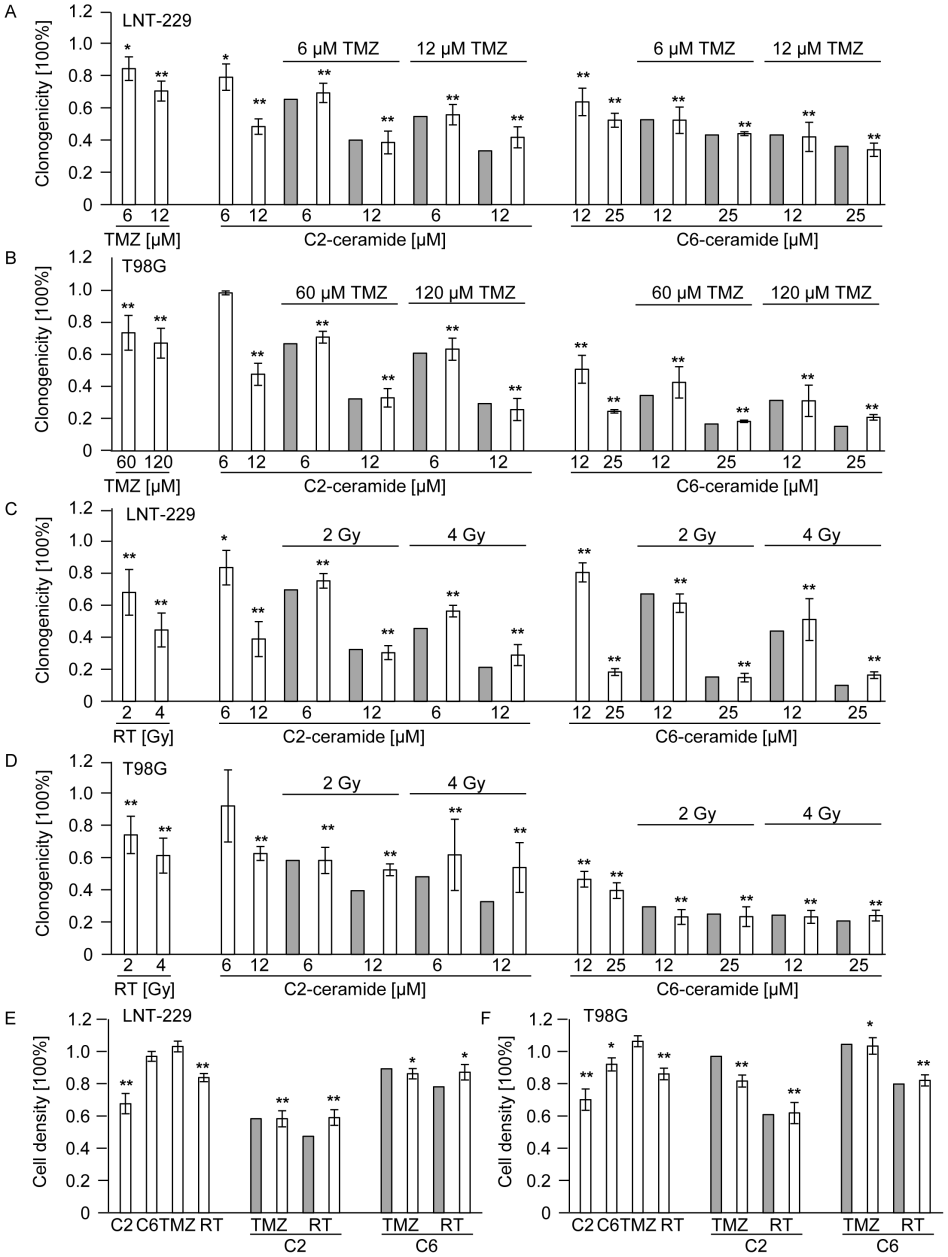
Co-treatment with exogenous ceramide or TMZ or irradiation shows additive but not synergistic effects in vitro. (A) LNT-229 or (B) T98G wt cells were treated with increasing concentrations of TMZ or C2- or C6-ceramide or co-treated with TMZ and C2- or C6-ceramide for 24 h and clonogenicity was assessed 2–3 weeks later. (C) LNT-229 or (D) T98G wt cells were treated with increasing concentrations of C2- or C6-ceramide for 24 h and increasing doses of irradiation (RT) or co-treated with C2- or C6-ceramide and irradiation and clonogenicity data were obtained 2–3 weeks later. (E) LNT-229 or (F) T98G wt cells were treated with a single dose of C2-ceramide (12 µM), C6-ceramide (12 µM) or TMZ (LNT-229: 12 µM; T98G: 125 µM) for 72 h or RT (8 Gy) or co-treated with C2- or C6-ceramide and TMZ or RT and cell density measured with crystal violet 72 h later. Predicted values, assessed using the fractional product method, are displayed in grey bars, observed values are highlighted by blank bars. Statistically significant changes compared to untreated cells are marked with asterisks (*, *p*<0.05; **, *p*<0.01; *t*-test).

To supplement the data listed above, short chain analogs of ceramide, C2- and C6-ceramide were used to mimic autocrine effects of endogenous ceramide. We examined the acute and long-term cytotoxic effects of C2- and C6-ceramide in LNT-229 and T98G glioblastoma cell lines. Both ceramide analogs induced acute cytotoxicity in a concentration-dependent manner (72 h treatment) (Fig. 6A, B). IC50 values for acute cytotoxicity were around 12 and 25 µM in both cell lines for C2-ceramide and around 25 and 50 µM for C6-ceramide. Lower concentrations of these ceramide analogs were sufficient to reduce clonogenic survival in human glioma cell lines, LNT-229 and T98G (Fig. 6C, D). Next, clonogenicity data were obtained when human glioma cells were co-treated with exogenous ceramide and TMZ (Fig. 7A, B) or irradiation (Fig. 7C, D). Again, no synergistic effects were observed in LNT-229 (Fig. 7A, C) or T98G cells (Fig. 7B, D) and the observed values matched the predicted values for independent activities. Single effects of TMZ, C2-ceramide, C6-ceramide or irradiation induced reduced clonogenicity in a concentration-/dose-dependent manner, as expected. Cell viability of LNT-229 (Fig. 7E) and T98G (Fig. 7F) cells was also assessed 72 h after treatment with a single dose of C2-ceramide, C6-ceramide, TMZ or irradiation. When short chain ceramide analogs were used in combination with TMZ or irradiation, no significant effects were observed either (Fig. 7E, F).

### Exogenous ceramide or PPMP induce acute cytotoxicity and reduce clonogenic survival in TMZ-resistant cell lines

**Figure 8:**
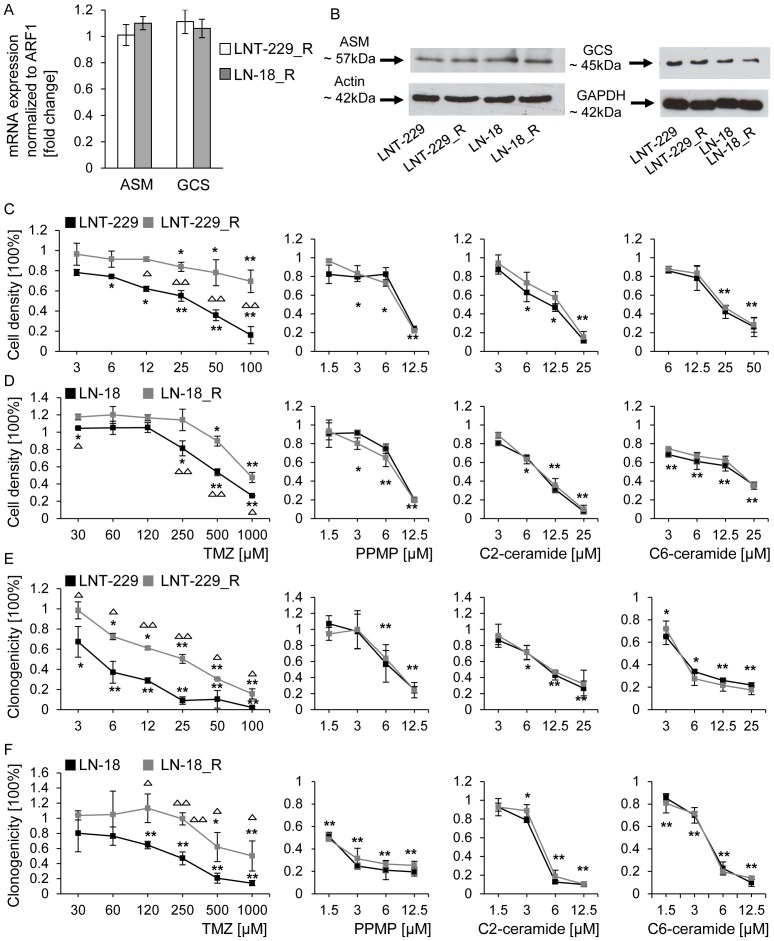
Exogenous ceramide and PPMP are cytotoxic to human glioma cell lines resistant to TMZ and reduce clonogenic survival. ASM and GCS (A) mRNA expression levels and (B) protein levels were assessed in LNT-229 and LN-18 parental and TMZ-resistant cell lines. Acute cytotoxicity in (C) LNT-229 and LNT-229_R and in (D) LN-18 and LN-18_R cell lines was assessed 72 h after treatment with increasing concentrations of TMZ, PPMP, C2-ceramide or C6-ceramide. For clonogenic survival data (E) LNT-229 and LNT-229_R or (F) LN-18 and LN-18_R cells were treated with increasing concentrations of TMZ, PPMP, C2-ceramide or C6-ceramide for 24 h, then medium was changed and cells were cultured for additional 2 weeks until colonies were detectable. Parental cells are highlighted by black squares, resistant cells are highlighted by grey squares. Statistically significant changes compared to untreated cells are marked with asterisks (*, *p*<0.05; **, *p*<0.01; *t*-test), statistically significant changes of parental to resistant cells are marked with open triangles (Δ, *p*<0.05; ΔΔ, *p*<0.01; *t*-test).

Finally, we investigated the effects of exogenous ceramide or PPMP in human glioma cell lines resistant to TMZ. Two cell lines, LNT-229 and LN-18, had been exposed repetitively to increasing concentrations of TMZ until a stable resistant phenotype (LNT-229_R and LN-18_R) was induced [41]. ASM and GCS mRNA and protein levels did not differ between parental and resistant cells (Fig. 8A, B). LNT-229 and LN-18 parental and resistant cells were then treated with increasing concentrations of TMZ. Significant differences between parental and resistant LNT-229 were found when cells were treated with TMZ in the range of 6–100 µM in an acute cytotoxicity assay (Fig. 8C) and in the range of 3–100 µM in a clonogenicity assay (Fig. 8E). For LN-18 parental and resistant cells, significant differences were observed for 30 µM and in the range of 250–1000 µM in an acute cytotoxicity assay (Fig. 8D) and in the range of 120–1000 µM in a clonogenicity assay (Fig. 8F). In a next step we assessed the cytotoxic effect of exogenous ceramide (C2-ceramide or C6-ceramide) or PPMP in parental and resistant cell lines. In both cell lines, LNT-229 and LN-18, exogenous ceramide as well as PPMP induced acute cytotoxicity (Fig. 8C, D) and decreased clonogenic survival (Fig. 8E, F) in a concentration-dependent manner, but without significant differences between parental and TMZ-resistant cells.

### Co-treatment with exogenous ceramide or PPMP and TMZ or irradiation shows additive but no synergistic effects in clonogenicity and acute cytotoxicity assays in TMZ-resistant glioma cells

**Figure 9:**
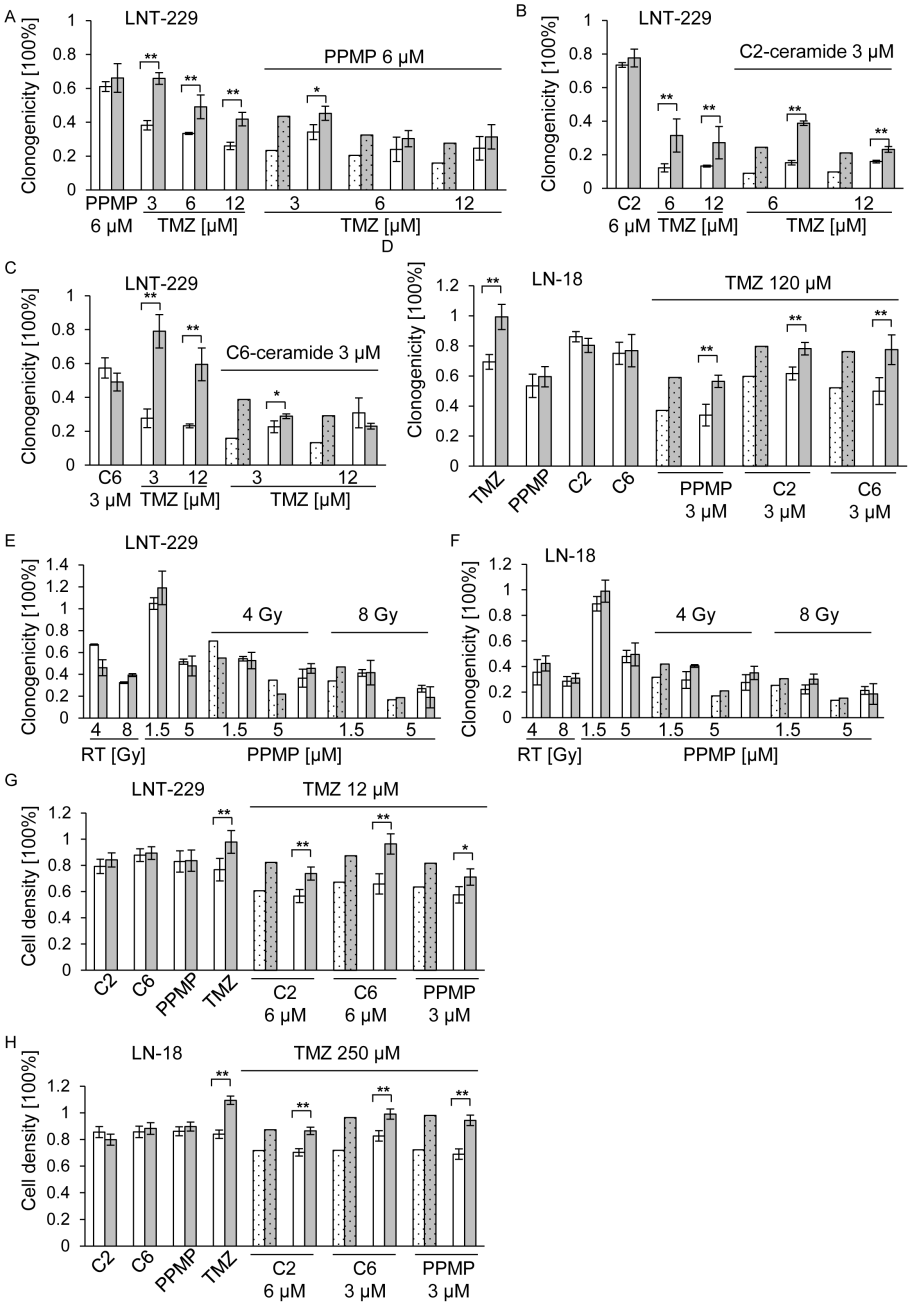
Co-treatment with exogenous ceramide or PPMP and TMZ or irradiation shows additive but no synergistic effects in clonogenicity and acute cytotoxicity assays in TMZ-resistant glioma cells. LNT-229 and LNT-229_R cells were treated with (A) PPMP or TMZ or co-treated with PPMP and TMZ or (B) C2-ceramide or TMZ or co-treated with C2-ceramide or (C) C6-ceramide or TMZ or co-treated with C6-ceramide and TMZ for 24 h, the medium was then changed and cells were cultured for additional 2 weeks until colonies were detectable. (D) LN-18 and LN-18_R cells were treated with PPMP or C2-ceramide or C6-ceramide or co-treated with PPMP or C2-ceramide or C6-ceramide for 24 h, the medium was then changed and cells were cultured for additional 2 weeks until colonies were detectable. (E) LNT-229 and LNT-229_R or (F) LN-18 and LN-18_R cells were treated with RT or PPMP or co-treated with PPMP for 24 h and RT. Clonogenicity data were assessed 2 weeks later colonies were detectable. (G) LNT-229 and LNT-229_R or (H) LN-18 and LN-18_R cells were treated with C2-ceramide, C6-ceramide, PPMP or TMZ alone or co-treated. Cell density was measured by crystal violet staining 72 h later. Parental cells are marked with white bars, resistant cells are marked with grey bars. Predicted values, assessed using the fractional product method, are highlighted in dotted bars, observed values are displayed by undotted bars. Statistically significant changes in resistant cells compared to wt cells are marked with asterisks (*, *p*<0.05; **, *p*<0.01; *t*-test).

Finally, we investigated whether co-treatment with PPMP or exogenous ceramide and TMZ could overcome resistance to TMZ in TMZ-resistant cells. Therefore LNT-229 and LNT-229_R cells were treated with a single concentration of PPMP (Fig. 9A) or C2-ceramide (Fig. 9B) or C6-ceramide (Fig. 9C) and different concentrations of TMZ for 24 h and were then cultured for additional 2 weeks until colony formation was detectable. The observed values matched with the predicted values for independent activities, in parental (as shown before) as well as in TMZ-resistant cell lines (Fig. 9A–C). The same occurred in LN-18 and LN-18_R cell lines (Fig. 9D), underscoring an additive but no synergistic effect of PPMP or exogenous ceramide in combination with TMZ, not only in parental but also in TMZ-resistant cell lines. To evaluate a possible synergy with irradiation in these TMZ-resistant cell lines, cells were irradiated and co-treated with PPMP, again demonstrating only additive effects in LNT-229 (Fig. 9E) and LN-18 (Fig. 9F) parental and resistant cells. To exclude that a synergistic effect would be detectable only in a short-time acute cytotoxicity assay, parental and TMZ-resistant cells were co-exposed to C2-ceramide, C6-ceramide or PPMP and TMZ for 72 h and cell density was measured using crystal violet staining in an acute cytotoxicity assay. When co-treatments were applied, however, the observed values in this acute cytotoxicity assay again matched the predicted values for independent actions for LNT-229 and LNT-299_R (Fig. 9G) as well as for LN-18 and LN-18_R (Fig.9H).

## Discussion

Ceramide is an important messenger initiating signal transduction pathways thought to promote tumor cell apoptosis in response to chemotherapy or irradiation [23], [30], [31], [32]. Several studies demonstrated that exogenous C2-ceramide induces apoptosis in almost any cell type, including human glioma cell lines [29]. However, the role of tumor cell endogenous ceramide levels for the primary resistance of glioma cells to the alkylating agents TMZ and CCNU as well as to radiation therapy requires a thorough investigation. We tested here whether modulation of ceramide levels in glioma cells themselves by ASM overexpression or PPMP treatment can overcome their resistance to TMZ, CCNU or irradiation. Surprisingly, modulations of intrinsic ceramide in human glioma cell lines trigger cytotoxicity, but do not synergistically enhance anti-tumor effects of TMZ or irradiation.

Initially, we reasoned that tumors with poor outcome should be characterized by low levels of the ceramide-generating enzyme ASM and high levels of the ceramide-catabolizing enzyme GCS. In fact, low levels of ASM were associated with a better outcome in glioblastoma patients although ASM expression appeared not to be dramatically altered in gliomas of different grades of malignancies (Fig. 1). GCS mRNA levels in glioblastomas were not different compared to normal brain tissue (Fig. 1E). Of note, GCS mRNA levels were significantly lower in astrocytomas WHO grade II/III, not only compared to glioblastoma, but also to normal brain (Fig. 1E). Yet, glioblastoma patients with higher GCS mRNA levels had inferior survival (Fig. 1H). Thus, increasing GSC mRNA correlates with ascending grades of malignancy, which may be reflected by low endogenous ceramide levels in high-grade gliomas [27] and increased GSC mRNA levels may characterize tumors that have a poor prognosis [35], [56].

We demonstrate that lentivirally induced ASM overexpression leads to increased endogenous ceramide levels and increased ASM activity in glioma cells, but does not affect proliferation (Fig. 2E), metabolic activity (Fig. 1F) or clonogenicity (Fig. 1G, H) of human glioma cells *in vitro*. Although it had been demonstrated that several stress stimuli resulted in ASM-induced ceramide accumulation leading to apoptosis [17], [18], [19], the stable ASM overexpression and induction of endogenous ceramide did not lead to sensitization of glioma cells to clinically relevant therapies (Fig. 3).

Interestingly, PPMP, a well-known GCS inhibitor [32] that promotes ceramide accumulation in human glioblastoma cell lines, induced cytotoxicity (Fig. 4C) and reduced clonogenicity (Fig. 4D). The cytotoxic effect of PPMP for glioblastomas is similar to previously published cytotoxic effects of this substance for colon cancer and breast cancer cells [57], [58]. Cytotoxic effects correlated directly to the employed PPMP in a concentration-dependent manner and particularly to the induced ceramide concentrations. These results were in accordance with the anti-glioma effects of exogenous ceramide (Fig. 6) but were difficult to reconcile with the failure of ASM expression to induce cell death or to sensitize for TMZ, CCNU or irradiation (Fig. 3). In the light of these findings the impression is supported that a very early selection step after attempted ASM overexpression may promote the survival of cells resistant to ASM and therefore ceramide induction. However, the incorporation of amitriptyline, a functional ASM inhibitor during lentiviral transduction, did not change the observed effects in functional assays. The observed lack of evidence for an effect of ASM overexpression on sensitization of glioma cells to therapy is paralleled by the worse survival probability of glioblastoma patients with high levels of ASM in the TCGA database (Fig. 1D).

Furthermore, the role of ASM overexpression or PPMP treatment for glioma resistance to current modalities of treatment was evaluated. Neither TMZ or CCNU nor irradiation led to synergistic anti-glioma effects in ASM-overexpressing LNT-229 or T98G (Fig. 3, Fig. 5), although the additional treatment regime produced increased ceramide release (data not shown). For other cell lines and other chemotherapeutic drugs, a sensitizing effect of ASM activation was described [20], [21], [22]. Moreover, previous reports have suggested that PPMP sensitized tumor cells to various therapeutic agents [59]. However, no synergistic anti-glioma activity was detected in our experiments applying co-treatments of PPMP and TMZ. Combined therapy with PPMP and either TMZ or irradiation led to additive rather than synergistic effects in both glioma cell lines tested. On the other hand, the endogenous ceramide levels induced by ASM overexpression or PPMP treatment may not have been in a dose range high enough to synergize with the added drug or additional irradiation effects. It seems also possible that there is a critical role of the ASM-ceramide system for chemotherapy-induced apoptosis, as shown for gemcitabine [32], but not for all therapeutic regimes, especially not for standard glioma treatments used for patients today. Alternatively, the subcellular localization of ceramide may determine the extent or type of damage that is induced.

Moreover, the application of exogenous ceramide induced apoptosis in human glioblastoma cells [29], resulting in cytotoxicity and reduced clonogenicity in our glioblastoma cell lines (Fig. 6), but we did not observe a synergistic enhancement of irradiation- or TMZ-mediated apoptosis (Fig. 7, Fig. 8). Yet, the addition of exogenous ceramide may mimic only some of the signaling aspects of endogenous ceramide. These findings, of course, do not exclude a role of the ASM-ceramide system in the tumor microenvironment, i.e. the non-malignant cells in the glioma microenvironment *in vivo*, for sensitizing to irradiation and/or alkylating agents.

In summary, efforts to modulate intrinsic ceramide levels by ASM overexpression or GCS inhibition by PPMP in glioma cells did not sensitize to alkylating chemotherapy or irradiation. Additionally, also exogenous ceramide applications did not result in synergistic effects with chemotherapeutics or irradiation in human glioma cells. These findings indicate that modulations of glioma cell intrinsic ceramide levels are not sufficient to overcome resistance to standard glioma therapies. We conclude that future studies on the role of modifications of ceramide in the glioma microenvironment for sensitization to irradiation or alkylating agents are warranted. This conclusion is supported by studies on experimental fibrosarcoma or melanoma in ASM-deficient mice. The tumors of these mice were resistant to radiation therapy *in vivo*. This phenotype was explained by the lack of radiation-induced ASM-mediated endothelial cell apoptosis *in vivo* [60] suggesting a role of host ASM for resistance to therapy. Thus, modulations of the ASM activity in the microenvironment *in vivo*, e.g. in the endothelial cell compartment, might be important for overcoming resistance to therapy in experimental gliomas.

## References

[1] Louis DN, Ohgaki H, Wiestler OD, Cavenee WK, Burger PC, et al (2007) The 2007 WHO classification of tumours of the central nervous system. Acta Neuropathol 114: 97–109. 17618441 10.1007/s00401-007-0243-4 PMC1929165

[2] Johnson DR, Ma DJ, Buckner JC, Hammack JE (2012) Conditional probability of long-term survival in glioblastoma: a population-based analysis. Cancer 118: 5608–5613. 22569786 10.1002/cncr.27590

[3] Stupp R, Mason WP, van den Bent MJ, Weller M, Fisher B, et al (2005) Radiotherapy plus concomitant and adjuvant temozolomide for glioblastoma. N Engl J Med 352: 987–996. 15758009 10.1056/NEJMoa043330

[4] Stupp R, Hegi ME, Mason WP, van den Bent MJ, Taphoorn MJ, et al (2009) Effects of radiotherapy with concomitant and adjuvant temozolomide versus radiotherapy alone on survival in glioblastoma in a randomised phase III study: 5-year analysis of the EORTC-NCIC trial. Lancet Oncol 10: 459–466. 19269895 10.1016/S1470-2045(09)70025-7

[5] Preusser M, de Ribaupierre S, Wohrer A, Erridge SC, Hegi M, et al (2011) Current concepts and management of glioblastoma. Ann Neurol 70: 9–21. 21786296 10.1002/ana.22425

[6] van den Bent MJ, Brandes AA, Rampling R, Kouwenhoven MC, Kros JM, et al (2009) Randomized phase II trial of erlotinib versus temozolomide or carmustine in recurrent glioblastoma: EORTC brain tumor group study 26034. J Clin Oncol 27: 1268–1274. 19204207 10.1200/JCO.2008.17.5984 PMC2667826

[7] Brada M, Stenning S, Gabe R, Thompson LC, Levy D, et al (2010) Temozolomide versus procarbazine, lomustine, and vincristine in recurrent high-grade glioma. J Clin Oncol 28: 4601–4608. 20855843 10.1200/JCO.2009.27.1932

[8] Weller M, Cloughesy T, Perry JR, Wick W (2013) Standards of care for treatment of recurrent glioblastoma-are we there yet? Neuro Oncol 15: 4–27. 23136223 10.1093/neuonc/nos273 PMC3534423

[9] Smith EL, Schuchman EH (2008) The unexpected role of acid sphingomyelinase in cell death and the pathophysiology of common diseases. FASEB J 22: 3419–3431. 18567738 10.1096/fj.08-108043 PMC2537423

[10] Senchenkov A, Litvak DA, Cabot MC (2001) Targeting ceramide metabolism–a strategy for overcoming drug resistance. J Natl Cancer Inst 93: 347–357. 11238696 10.1093/jnci/93.5.347

[11] Gulbins E, Grassme H (2002) Ceramide and cell death receptor clustering. Biochim Biophys Acta 1585: 139–145. 12531547 10.1016/s1388-1981(02)00334-7

[12] Grassme H, Riethmuller J, Gulbins E (2007) Biological aspects of ceramide-enriched membrane domains. Prog Lipid Res 46: 161–170. 17490747 10.1016/j.plipres.2007.03.002

[13] Cifone MG, De Maria R, Roncaioli P, Rippo MR, Azuma M, et al (1994) Apoptotic signaling through CD95 (Fas/Apo-1) activates an acidic sphingomyelinase. J Exp Med 180: 1547–1552. 7523573 10.1084/jem.180.4.1547 PMC2191710

[14] Brenner B, Ferlinz K, Grassme H, Weller M, Koppenhoefer U, et al (1998) Fas/CD95/Apo-I activates the acidic sphingomyelinase via caspases. Cell Death Differ 5: 29–37. 10200443 10.1038/sj.cdd.4400307

[15] Gulbins E, Bissonnette R, Mahboubi A, Martin S, Nishioka W, et al (1995) FAS-induced apoptosis is mediated via a ceramide-initiated RAS signaling pathway. Immunity 2: 341–351. 7536620 10.1016/1074-7613(95)90142-6

[16] Schutze S, Potthoff K, Machleidt T, Berkovic D, Wiegmann K, et al (1992) TNF activates NF-kappa B by phosphatidylcholine-specific phospholipase C-induced “acidic” sphingomyelin breakdown. Cell 71: 765–776. 1330325 10.1016/0092-8674(92)90553-o

[17] Pena LA, Fuks Z, Kolesnick RN (2000) Radiation-induced apoptosis of endothelial cells in the murine central nervous system: protection by fibroblast growth factor and sphingomyelinase deficiency. Cancer Res 60: 321–327. 10667583

[18] Santana P, Pena LA, Haimovitz-Friedman A, Martin S, Green D, et al (1996) Acid sphingomyelinase-deficient human lymphoblasts and mice are defective in radiation-induced apoptosis. Cell 86: 189–199. 8706124 10.1016/s0092-8674(00)80091-4

[19] Chatterjee M, Wu S (2001) Involvement of Fas receptor and not tumor necrosis factor-alpha receptor in ultraviolet-induced activation of acid sphingomyelinase. Mol Carcinog 30: 47–55. 11255263 10.1002/1098-2744(200101)30:1<47::aid-mc1012>3.0.co;2-3

[20] Lacour S, Hammann A, Grazide S, Lagadic-Gossmann D, Athias A, et al (2004) Cisplatin-induced CD95 redistribution into membrane lipid rafts of HT29 human colon cancer cells. Cancer Res 64: 3593–3598. 15150117 10.1158/0008-5472.CAN-03-2787

[21] Prinetti A, Millimaggi D, D'Ascenzo S, Clarkson M, Bettiga A, et al (2006) Lack of ceramide generation and altered sphingolipid composition are associated with drug resistance in human ovarian carcinoma cells. Biochem J 395: 311–318. 16356169 10.1042/BJ20051184 PMC1422777

[22] Lovat PE, Di Sano F, Corazzari M, Fazi B, Donnorso RP, et al (2004) Gangliosides link the acidic sphingomyelinase-mediated induction of ceramide to 12-lipoxygenase-dependent apoptosis of neuroblastoma in response to fenretinide. J Natl Cancer Inst 96: 1288–1299. 15339967 10.1093/jnci/djh254

[23] Grammatikos G, Teichgraber V, Carpinteiro A, Trarbach T, Weller M, et al (2007) Overexpression of acid sphingomyelinase sensitizes glioma cells to chemotherapy. Antioxid Redox Signal 9: 1449–1456. 17576160 10.1089/ars.2007.1673

[24] Hara S, Nakashima S, Kiyono T, Sawada M, Yoshimura S, et al (2004) p53-Independent ceramide formation in human glioma cells during gamma-radiation-induced apoptosis. Cell Death Differ 11: 853–861. 15088070 10.1038/sj.cdd.4401428

[25] Selzner M, Bielawska A, Morse MA, Rudiger HA, Sindram D, et al (2001) Induction of apoptotic cell death and prevention of tumor growth by ceramide analogues in metastatic human colon cancer. Cancer Res 61: 1233–1240. 11221856

[26] Rylova SN, Somova OG, Dyatlovitskaya EV (1998) Comparative investigation of sphingoid bases and fatty acids in ceramides and sphingomyelins from human ovarian malignant tumors and normal ovary. Biochemistry (Mosc) 63: 1057–1060. 9795275

[27] Riboni L, Campanella R, Bassi R, Villani R, Gaini SM, et al (2002) Ceramide levels are inversely associated with malignant progression of human glial tumors. Glia 39: 105–113. 12112362 10.1002/glia.10087

[28] Giussani P, Bassi R, Anelli V, Brioschi L, De Zen F, et al (2012) Glucosylceramide synthase protects glioblastoma cells against autophagic and apoptotic death induced by temozolomide and Paclitaxel. Cancer Invest 30: 27–37. 22236187 10.3109/07357907.2011.629379

[29] Wagenknecht B, Roth W, Gulbins E, Wolburg H, Weller M (2001) C2-ceramide signaling in glioma cells: synergistic enhancement of CD95-mediated, caspase-dependent apoptosis. Cell Death Differ 8: 595–602. 11536010 10.1038/sj.cdd.4400848

[30] Gouaze V, Yu JY, Bleicher RJ, Han TY, Liu YY, et al (2004) Overexpression of glucosylceramide synthase and P-glycoprotein in cancer cells selected for resistance to natural product chemotherapy. Mol Cancer Ther 3: 633–639. 15141021

[31] Liu YY, Han TY, Giuliano AE, Cabot MC (1999) Expression of glucosylceramide synthase, converting ceramide to glucosylceramide, confers adriamycin resistance in human breast cancer cells. J Biol Chem 274: 1140–1146. 9873062 10.1074/jbc.274.2.1140

[32] Dumitru CA, Weller M, Gulbins E (2009) Ceramide metabolism determines glioma cell resistance to chemotherapy. J Cell Physiol 221: 688–695. 19711353 10.1002/jcp.21907

[33] Maurer BJ, Metelitsa LS, Seeger RC, Cabot MC, Reynolds CP (1999) Increase of ceramide and induction of mixed apoptosis/necrosis by N-(4-hydroxyphenyl)- retinamide in neuroblastoma cell lines. J Natl Cancer Inst 91: 1138–1146. 10393722 10.1093/jnci/91.13.1138

[34] Maurer BJ, Melton L, Billups C, Cabot MC, Reynolds CP (2000) Synergistic cytotoxicity in solid tumor cell lines between N-(4-hydroxyphenyl)retinamide and modulators of ceramide metabolism. J Natl Cancer Inst 92: 1897–1909. 11106681 10.1093/jnci/92.23.1897

[35] Xie P, Shen YF, Shi YP, Ge SM, Gu ZH, et al (2008) Overexpression of glucosylceramide synthase in associated with multidrug resistance of leukemia cells. Leuk Res 32: 475–480. 17709137 10.1016/j.leukres.2007.07.006

[36] Ogretmen B, Hannun YA (2004) Biologically active sphingolipids in cancer pathogenesis and treatment. Nat Rev Cancer 4: 604–616. 15286740 10.1038/nrc1411

[37] Veldman RJ, Mita A, Cuvillier O, Garcia V, Klappe K, et al (2003) The absence of functional glucosylceramide synthase does not sensitize melanoma cells for anticancer drugs. FASEB J 17: 1144–1146. 12692077 10.1096/fj.02-1053fje

[38] Tepper AD, Diks SH, van Blitterswijk WJ, Borst J (2000) Glucosylceramide synthase does not attenuate the ceramide pool accumulating during apoptosis induced by CD95 or anti-cancer regimens. J Biol Chem 275: 34810–34817. 10945987 10.1074/jbc.M005142200

[39] Shayman JA, Lee L, Abe A, Shu L (2000) Inhibitors of glucosylceramide synthase. Methods Enzymol 311: 373–387. 10563341 10.1016/s0076-6879(00)11097-3

[40] Tabatabai G, Frank B, Wick A, Lemke D, von Kurthy G, et al (2007) Synergistic antiglioma activity of radiotherapy and enzastaurin. Ann Neurol 61: 153–161. 17212356 10.1002/ana.21057

[41] Happold C, Roth P, Wick W, Schmidt N, Florea AM, et al (2012) Distinct molecular mechanisms of acquired resistance to temozolomide in glioblastoma cells. J Neurochem 122: 444–455. 22564186 10.1111/j.1471-4159.2012.07781.x

[42] Demaison C, Parsley K, Brouns G, Scherr M, Battmer K, et al (2002) High-level transduction and gene expression in hematopoietic repopulating cells using a human immunodeficiency [correction of imunodeficiency] virus type 1-based lentiviral vector containing an internal spleen focus forming virus promoter. Hum Gene Ther 13: 803–813. 11975847 10.1089/10430340252898984

[43] Tabatabai G, Hasenbach K, Herrmann C, Maurer G, Mohle R, et al (2010) Glioma tropism of lentivirally transduced hematopoietic progenitor cells. Int J Oncol 36: 1409–1417. 20428764 10.3892/ijo_00000626

[44] Hsiao LL, Dangond F, Yoshida T, Hong R, Jensen RV, et al (2001) A compendium of gene expression in normal human tissues. Physiol Genomics 7: 97–104. 11773596 10.1152/physiolgenomics.00040.2001

[45] Pfaffl MW (2001) A new mathematical model for relative quantification in real-time RT-PCR. Nucleic Acids Res 29: e45. 11328886 10.1093/nar/29.9.e45 PMC55695

[46] Roth W, Grimmel C, Rieger L, Strik H, Takayama S, et al (2000) Bag-1 and Bcl-2 gene transfer in malignant glioma: modulation of cell cycle regulation and apoptosis. Brain Pathol 10: 223–234. 10764042 10.1111/j.1750-3639.2000.tb00256.x PMC8098428

[47] Gloeckner H, Jonuleit T, Lemke HD (2001) Monitoring of cell viability and cell growth in a hollow-fiber bioreactor by use of the dye Alamar Blue. J Immunol Methods 252: 131–138. 11334972 10.1016/s0022-1759(01)00347-7

[48] Madhavan S, Zenklusen JC, Kotliarov Y, Sahni H, Fine HA, et al (2009) Rembrandt: helping personalized medicine become a reality through integrative translational research. Mol Cancer Res 7: 157–167. 19208739 10.1158/1541-7786.MCR-08-0435 PMC2645472

[49] McLendon R, Friedman A, Bigner D, Van Meir E, Brat D, et al (2008) Comprehensive genomic characterization defines human glioblastoma genes and core pathways. Nature 455: 1061–1068. 18772890 10.1038/nature07385 PMC2671642

[50] Becker KA, Riethmuller J, Luth A, Doring G, Kleuser B, et al (2010) Acid sphingomyelinase inhibitors normalize pulmonary ceramide and inflammation in cystic fibrosis. Am J Respir Cell Mol Biol 42: 716–724. 19635928 10.1165/rcmb.2009-0174OC

[51] Eberhardt O, Coelln RV, Kugler S, Lindenau J, Rathke-Hartlieb S, et al (2000) Protection by synergistic effects of adenovirus-mediated X-chromosome-linked inhibitor of apoptosis and glial cell line-derived neurotrophic factor gene transfer in the 1-methyl-4-phenyl-1,2,3,6-tetrahydropyridine model of Parkinson's disease. J Neurosci 20: 9126–9134. 11124990 10.1523/JNEUROSCI.20-24-09126.2000 PMC6773033

[52] Greco WR, Bravo G, Parsons JC (1995) The search for synergy: a critical review from a response surface perspective. Pharmacol Rev 47: 331–385. 7568331

[53] Ferlinz K, Hurwitz R, Vielhaber G, Suzuki K, Sandhoff K (1994) Occurrence of two molecular forms of human acid sphingomyelinase. Biochem J 301 (Pt 3): 855–862. 10.1042/bj3010855 PMC1137065 8053910

[54] Hermisson M, Klumpp A, Wick W, Wischhusen J, Nagel G, et al (2006) O6-methylguanine DNA methyltransferase and p53 status predict temozolomide sensitivity in human malignant glioma cells. J Neurochem 96: 766–776. 16405512 10.1111/j.1471-4159.2005.03583.x

[55] Wick A, Wick W, Hirrlinger J, Gerhardt E, Dringen R, et al (2004) Chemotherapy-induced cell death in primary cerebellar granule neurons but not in astrocytes: in vitro paradigm of differential neurotoxicity. J Neurochem 91: 1067–1074. 15569250 10.1111/j.1471-4159.2004.02774.x

[56] Liu YY, Patwardhan GA, Xie P, Gu X, Giuliano AE, et al (2011) Glucosylceramide synthase, a factor in modulating drug resistance, is overexpressed in metastatic breast carcinoma. Int J Oncol 39: 425–431. 21617856 10.3892/ijo.2011.1052 PMC4037863

[57] Boyle PJ, Ma R, Tuteja N, Banerjee S, Basu S (2006) Apoptosis of human breast carcinoma cells in the presence of cis-platin and L-/D-PPMP: IV. Modulation of replication complexes and glycolipid: Glycosyltransferases. Glycoconj J 23: 175–187. 16691501 10.1007/s10719-006-7923-5

[58] Basu S, Ma R, Boyle PJ, Mikulla B, Bradley M, et al (2004) Apoptosis of human carcinoma cells in the presence of potential anti-cancer drugs: III. Treatment of Colo-205 and SKBR3 cells with: cis-platin, tamoxifen, melphalan, betulinic acid, L-PDMP, L-PPMP, and GD3 ganglioside. Glycoconj J 20: 563–577. 15454695 10.1023/B:GLYC.0000043293.46845.07

[59] Liu YY, Han TY, Giuliano AE, Cabot MC (2001) Ceramide glycosylation potentiates cellular multidrug resistance. FASEB J 15: 719–730. 11259390 10.1096/fj.00-0223com

[60] Garcia-Barros M, Lacorazza D, Petrie H, Haimovitz-Friedman A, Cardon-Cardo C, et al (2004) Host acid sphingomyelinase regulates microvascular function not tumor immunity. Cancer Res 64: 8285–8291. 15548696 10.1158/0008-5472.CAN-04-2715

